# Differential expression of 10 genes in the hypothalamus of two generations of rats selected for a reaction to humans

**DOI:** 10.18699/VJ21.50-o

**Published:** 2021-03

**Authors:** N.V. Klimova, I.V. Chadaeva, S.G. Shichevich, R.V. Kozhemyakina

**Affiliations:** Institute of Cytology and Genetics of Siberian Branch of the Russian Academy of Sciences, Novosibirsk, Russia; Institute of Cytology and Genetics of Siberian Branch of the Russian Academy of Sciences, Novosibirsk, Russia; Institute of Cytology and Genetics of Siberian Branch of the Russian Academy of Sciences, Novosibirsk, Russia; Institute of Cytology and Genetics of Siberian Branch of the Russian Academy of Sciences, Novosibirsk, Russia

**Keywords:** aggressive behavior, tame behavior, gene expression, hypothalamus, rats, агрессивное и ручное поведение, дифференциальная экспрессия генов, гипоталамус, крысы

## Abstract

Individual behavioral differences are due to an interaction of the genotype and the environment. Phenotypic manifestation of aggressive behavior depends on the coordinated expression of gene ensembles. Nonetheless, the identification of these genes and of combinations of their mutual influence on expression remains a difficult task. Using animal models of aggressive behavior (gray rats that were selected for a reaction to humans; tame and aggressive rat strains), we evaluated the expression of 10 genes potentially associated with aggressiveness according to the literature: Cacna1b, Cacna2d3, Drd2, Egr1, Gad2, Gria2, Mapk1, Nos1, Pomc, and Syn1. To identify the genes most important for the manifestation of aggressiveness, we analyzed the expression of these genes in two generations of rats: 88th and 90th. Assessment of gene expression levels was carried out by real-time PCR in the hypothalamus of tame and aggressive rats. This analysis confirmed that 4 out of the 10 genes differ in expression levels between aggressive rats and tame rats in both generations. Specifically, it was shown that the expression of the Cacna1b, Drd2, Egr1, and Gad2 genes does not differ between the two generations (88th vs 90th) within each strain, but significantly differs between the strains: in the tame rats of both generations, the expression levels of these genes are significantly lower as compared to those in the aggressive rats. Therefore, these genes hold promise for further studies on behavioral characteristics. Thus, we confirmed polygenic causes of phenotypic manifestation of aggressive reactions.

## Abstract (ru)

Индивидуальные особенности поведения у особей одного вида обусловлены взаимодействием генотипа и социального опыта. Как у любого типа поведения, фенотипическое проявление паттернов агрессивного поведения зависит от согласованной экспрессии целых ансамблей генов. Однако идентификация этих генов и комбинаций их взаимного влияния на экспрессию остается сложной задачей. С целью выявления наиболее значимых для осуществления агрессивных реакций генов нами на модельных животных – серых крысах, селекционируемых по реакции на человека (линии ручных и агрессивных крыс), была проведена оценка уровня экспрессии выбранных на основе литературных данных десяти генов (Cacna1b, Cacna2d3, Drd2, Egr1, Gad2, Gria2, Mapk1, Nos1, Pomc, Syn1), которые ассоциированы с агрессивным поведением. Экспрессию генов оценивали методом ПЦР в реальном времени в образцах гипоталамуса ручных и агрессивных серых крыс двух разных поколений (88-е и 90-е). В результате проведенного анализа экспрессии генов в гипоталамусе крыс, селекционируемых на ручное и агрессивное поведение, было обнаружено, что четыре из десяти исследуемых генов достоверно различаются по уровню экспрессии между крысами агрессивной и ручной линий 88-го и 90-го поколений разведения. Кроме того, показано, что экспрессия генов Cacna1b, Drd2, Egr1 и Gad2 не изменяется между двумя поколениями крыс одной и той же линии, но достоверно различается между линиями: у крыс ручной линии обоих поколений эти гены экспрессируются достоверно ниже по сравнению с агрессивной. Гены Cacna1b, Drd2, Egr1 и Gad2 являются наиболее перспективными для дальнейших исследований поведенческих особенностей крыс, селекционируемых по реакции на человека. Данный результат подтверждает полигенную детерминацию фенотипического проявления агрессивных реакций на примере модельных животных

## Introduction

Behavioral patterns in individuals of the same species are due to the interaction of a genotype and social experience (Lindenfors, Tullberg, 2011; Anholt, Mackay, 2012; Kudryavtseva et al., 2014; Markel, 2016). At the same time, it is difficult to identify genes associated with a specific behavior type and combinations of their mutual influence on each other. Studies on aggressive behavior and its genetic causation (i. e., regulation of aggressive reactions) require experiments on model animals that differ in some aggressiveness parameter, so that it is possible to adequately assess the phenotypic manifestations of aggressiveness under the conditions that are set up and controlled by researchers (VanOortmerssen, Bakker, 1981; Kudryavtseva et al., 2014). Experimental studies on model animals will make it possible to identify orthologous genes associated with aggressive behavior in different species; these data are necessary for subsequent identification of evolutionary patterns in how aggressiveness is determined by genetic factors in animals.

It is known that the level of aggressiveness is inherited; genetic control of the phenotypic variation in the aggressiveness level in animal populations has been confirmed experimentally (VanOortmerssen, Bakker, 1981; Hudziak et al., 2003; Fairbanks et al., 2004; Saetre et al., 2006). Most of such studies are focused on one specific gene out of those associated with aggressive behavior, for example, studies on the differential expression of genes of the estrogen receptor (Cushing, 2016), serotonin receptor (Cervantes, Delville, 2009; Naumenko et al., 2009), dopamine receptor (Golden et al., 2019), Maoa (Chu et al., 2017), genes Bdnf (Ilchibaeva et al., 2015) and Nos1 (Wultsch et al., 2007), and other well-known genes associated with aggressiveness.

On the other hand, many reviews on the genetics of aggressive behavior indicate polygenic causes of aggressive behavior in animals, i. e., phenotypic manifestation of individual aggressive reactions is controlled by simultaneous expression of many genes, namely, whole ensembles of genes (Craig, Halton, 2009; Anholt, Mackay, 2012; Pavlov et al., 2012; Kudryavtseva et al., 2014; Hoopfer, 2016; Markel, 2016).

In rats of tame and aggressive strains, the expression of gene groups in cerebral hemispheres of males and females has been investigated (Albert et al., 2012), but there are some difficulties with correct interpretation of the results because there are known effect of the ovulation cycle on all physiological processes of the female body. In another work, differentially expressed genes were revealed in hybrid animals of the 2nd generation, obtained by crossing tame and aggressive rats (Heyne et al., 2014). Undoubtedly, cerebral hemispheres play a leading role in the implementation of higher brain functions. Nonetheless, genetic control of aggressive behavioral reactions is primarily carried out by the hypothalamus: the central brain structure that controls emotions. Studies have shown that electrical stimulation of some areas of the hypothalamus leads to the manifestation of aggressive behavior (Kruk, 1991; Hrabovszky et al., 2005; Lin et al., 2011).

Therefore, in our work, we analyzed expression levels of 10 genes in the hypothalamus, those that, according to the literature, are associated with aggressive behavior. For this purpose, we used model animals, rats, while tracing the stability of gene expression in two generations of the studied rats.

Namely, we used males of two outbred strains of gray rats (Rattus norvegicus). The rats had been selected for elimination (tame or domesticated) and enhancement of aggressivedefensive reaction to humans (aggressive, respectively; Belyaev, Borodin, 1985; Plyusnina et al., 2007). In response to the presentation of the stimulus, i. e., a researcher’s hand in a thick glove (this procedure is called the “glove test”), the rats of the tame strain reacted calmly, i. e., approached and sniffed the glove without performing any aggressive actions; on the contrary, the rats of the aggressive strain reacted violently by immediately attacking the stimulus. Tame and aggressive rats were taken from 88th and 90th generations of breeding. Studies of the tame and aggressive rats after 60–70 generations have shown differences in some behavioral reactions in the open field test, Morris water maze test, and elevated plus maze test as well as differences in morphometric parameters of the cranium and changes in fur coloration (Plyusnina et al., 2007; Kozhemyakina et al., 2016; Kozhemyakina, 2017).

Expression levels of 10 genes were analyzed:

- Cacna1b (calcium voltage-gated channel subunit alpha1B) and Cacna2d3 (calcium voltage-gated channel auxiliary subunit alpha2delta3) encode subunits of highthreshold calcium channels that release neurotransmitters. Calcium channels play a critical part in the manifestation of aggressive behavior through synaptic transmission of neurotransmitters GABA and serotonin (Kim C. et al., 2009).
- The Drd2 gene (dopamine receptor D2) is the gene for dopamine receptor D2, which is involved in the processes of motivation and learning; changes in the expression of the Drd2 gene cause various pathologies, including increased aggressiveness (Miczek et al., 2002; Kim V. et al., 2015).
- The Egr1 gene (early growth response 1) encodes a protein that activates the transcription of genes participating in cell division and differentiation. Egr1 is a transcription factor that regulates the expression of several genes that are associated with long-term memory (Knapska, Kaczmarek, 2004). It is known that Egr1 expression increases in response to stress (Knapska, Kaczmarek, 2004; Hodges et al., 2014), and, in addition, Egr1 knockout male mice do not demonstrate aggressive behavior in the presence of other males (Topilko et al., 1998).
- The Gad2 gene (glutamate decarboxylase 2) encodes glutamate decarboxylase, which catalyzes the conversion of glutamate to GABA (a neurotransmitter that inhibits neuronal electrical impulses), and thus the Gad2 gene takes part in the control of the emotional state of experimental animals, by regulating social, including aggressive, behavior (Stork et al., 2000). In particular, it has been reported that Gad2 knockout mice have lower levels of aggressive-behavior indicators
- The Gria2 gene (glutamate ionotropic receptor AMPA type subunit 2) encodes a subunit of glutamate receptor: the most important participant of excitatory processes in the central nervous system. Blockage of this receptor in naive mice decreases aggressiveness in comparison with littermates having normally functioning glutamate receptors (Vekovischeva et al., 2004).
- The Mapk1 gene (mitogen-activated protein kinase 1) encodes a mitogen-activated protein kinase, which performs a complex function in cellular processes (e.g., control of gene transcription, metabolism, and proliferation) in central-nervous-system neurons. It was demonstrated that mice with a conditional knockout of this gene exhibit increased aggressiveness (Satoh et al., 2011).
- The Nos1 gene (nitric oxide synthase 1) encodes an enzyme, neuronal nitric oxide synthase, that catalyzes the synthesis of nitric oxide and is an important player in neurotransmission. Studies have shown that the role of the Nos1 gene in aggressive behavior is based on the interaction of nitric oxide synthase with serotonin transporter, and this process decreases serotonin uptake (Nelson et al., 1995; Reif et al., 2009; Veroude et al., 2016) and leads to a decrease in aggressiveness (Kulikov et al., 2012).
- The Pomc gene (proopiomelanocortin) is a gene of a prohormone, proopiomelanocortin, which is a precursor of adrenocorticotropic hormone. Studies have revealed that melanocortin is associated with aggressive behavior (Værøy et al., 2018). In particular, in aggressive foxes, the level of expression of the Pomc gene is lower as compared to tame foxes (Gulevich et al., 2004).
- The Syn1 gene (synapsin I) encodes a phosphoprotein that regulates the release of neurotransmitters in synapses on the surface of synaptic vesicles. Research on rats and mice indicates a decrease in the expression of Syn1 during chronic stress and early isolation (Elizalde et al., 2010; Park et al., 2014), which is usually accompanied by changes of behavior in general and aggressiveness in particular.

## Materials and methods

**Experimental animals.** The number of experimental rats was determined and experiments on the rats were carried out in accordance with international European bioethical standards (Directive 2010/63/EU) and the Guidelines for the Care and Use of Laboratory Animals approved by the Ministry of Health of Russia (Appendix to decree No. 267 of June 19, 2003).

The work was performed on sexually mature males of the 88th and 90th generations of two outbred strains (tame and aggressive). The experiment involved 6 animals ofthe 88th generation (3 tame rats vs. 3 aggressive rats) and 12 animals from the 90th generation (6 tame rats vs. 6 aggressive rats). To exclude the influence of the photoperiod on the physiology and behavior of the experimental animals, we used rats born at the same time of the year. In accordance with the selection criterion (a reaction to humans in the glove test; Belyaev, Borodin, 1985; Plyusnina et al., 2007), the aggressive-defensive response in selected aggressive rats corresponded to a score of –3.5 points. For tame rats, the behavioral score in the glove test was +3.5 points, which is an indicator of strong domestication.

**Isolation of total RNA and real-time PCR (RT-PCR).** Hypothalamic samples were dissected postmortem, collected into liquid nitrogen, and stored at –70 °C until use. Total RNA was extracted from frozen tissue specimens using the TRIzol™ Reagent (Invitrogen, USA) according to the manufacturer’s protocol. RNA quality was evaluated on an Invitrogen Qubit™ 2.0 fluorometer (Invitrogen/Life Technologies, USA). The RNA was purified using paramagnetic RNAClean XP beads (Beckman Coulter, USA) and dissolved in double-distilled water. To remove impurities of genomic DNA, the RNA was treated with DNase I (Thermo Fisher Scientific, USA). RNA quality was determined on Agilent Bioanalyzer 2100 (Agilent, Santa-Clara, CA, USA).

Complementary DNA (cDNA) was synthesized with kits from Syntol (Russia). The reaction included 1 μg of RNA, and all the procedures were carried out according to the manufacturer’s protocols. Oligonucleotide primers for RT-PCR were designed in the PrimerBLAST software (see the Table). Gene expression was assessed by RT-PCR using the CFX96 RealTime PCR Detection System (Bio-Rad, USA). After the PCR, for reactions with the intercalating dye EVAGreen, product specificity was assessed by melting-curve analysis. Each reaction was carried out in duplicate (technical replicates). Amplification efficiency was 90 to 110 % for each primer pair. Target genes’ expression values were normalized to Rpl30 expression as a reference.

**Table 1.**
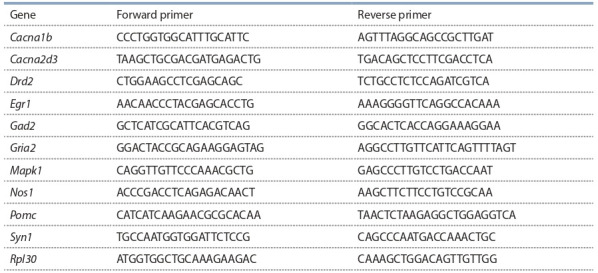
Primer sequences for RT-PCR (5’→3’)

**Statistical analysis.** This analysis of the PCR results was performed by Student’s t test as well as factor analysis (multivariate exploratory techniques: factor analysis, varimax, and variance maximization). The statistical analyses were performed in Statistica 6.0. Results are presented as mean ± standard error of the mean, and data satisfying the condition p < 0.05 were considered statistically significant.

## Results

**Fig. 1.**
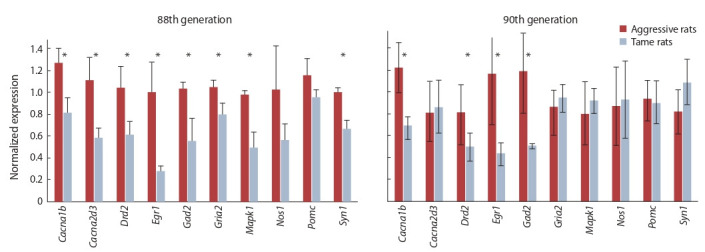
Normalized Cacna1b, Cacna2d3, Drd2, Egr1, Gad2, Gria2, Mapk1, Nos1, Pomc, and Syn1 mRNA levels in the hypothalamus of tame and aggressive rats of the 88th and 90th generations. Data are presented as mean±standard error of the mean. The significance of the interstrain differences is indicated by an asterisk (p < 0.05)

By RT-PCR verification in the hypothalamus of 88th generation rats, genes were identified that were differentially expressed between the aggressive strain and tame strain of rats. Thus, in aggressive rats, expression levels of genes Cacna1b, Cacna2d3, Drd2, Egr1, Gad2, Gria2, Mapk1, and Syn1 were found to be significantly higher as compared to tame rats (Fig. 1; t test p < 0.05). The expression of genes Nos1 and Pomc did not differ significantly between tame and aggressive rats of the 88th generation of the selection for the reaction to humans

The expression of genes Cacna1b, Drd2, Egr1, and Gad2 in the hypothalamus turned out to be significantly higher in aggressive 90th generation rats than in tame rats of the same generation (see Fig. 1; p < 0.05). On the contrary, in these animals, no significant interstrain differences were found in the expression of genes Cacna2d3, Gria2, Mapk1, Nos1, Pomc, and Syn1.

In the assay of mRNA levels of the same genes in the hypothalamic samples from rats of the 88th and 90th generations, it was found that the expression of Cacna1b, Drd2, Egr1, and Gad2 is significantly lower in rats of the tame strain than in the aggressive strain, regardless of the generation. Therefore, these genes hold promise for further research as genes determining the behavioral phenotype of rats during the selection for the reaction to humans.

**Fig. 2.**
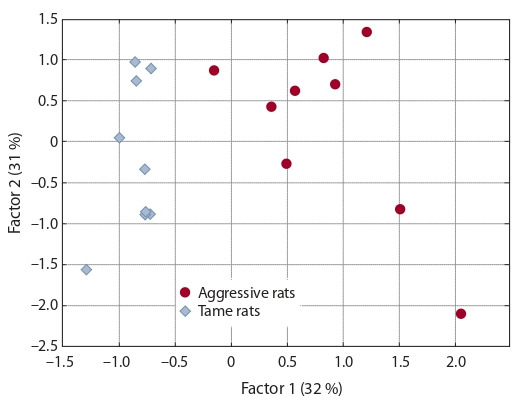
Significant factors of genetic variability of the studied genes’ expression in aggressive and tame rats, as revealed by the Varimax method with standard parameters of the Statistica 6.0 software.

Additionally, in the factor analysis of the pooled data on gene expression in animals of the 88th and 90th generations, only two significant factors were identified (Fig. 2). The first factor significantly correlates (p < 0.05, Student’s t test) with the expression of 4 genes (Cacna1b: linear correlation coefficient r = 0.94, Drd2: r = 0.77, Egr1: r = 0.92, and Gad2: r = 0.85) and explains the percentage of variance (32 %) in the experimental data that corresponds to the difference between aggressive and tame rats. The second factor significantly correlates with the expression of 3 other genes (Cacna2d3: r = 0.91, Gria2: r = 0.92, and Mapk1: r = 0.93) and indicates intragroup variance (31 %) common between the aggressive and tame animals. The third factor accounts for 12 % of the variance but does not significantly correlate with the expression of any analyzed genes (data not shown).

## Discussion

Here, in our analysis of RT-PCR data, between tame and aggressive rats (two generations: 88th and 90th generations of rats selected for a reaction to humans), we identified 4 differentially expressed genes (Cacna1b, Drd2, Egr1, and Gad2) out of the 10 studied. Meanwhile, it was found that mRNA levels of these genes do not differ between the two generations within each strain.

The Cacna1b gene encodes the Cav2.2 protein, which is a subunit of high-threshold calcium channels that control the release of neurotransmitters from neurons. This subunit of the calcium channel regulates the passage of calcium ions, thereby determining the properties of the channel. The Cacna1b gene is expressed weakly in the brain (Castiglioni et al., 2006), but the calcium channel subunit encoded by it plays an important role in the body’s response to aversive stimuli (Bunda et al., 2019). Calcium channels promote a release of neurotransmitters at excitatory synapses, resulting in suppression of exploratory behavior on the one hand and novelty-induced anxiety-like behavior (Bunda et al., 2019) on the other. Nevertheless, as demonstrated in the 74th generation of rats selected for a reaction to humans, the exploratory behavior in the open field test is practically the same between tame and aggressive rats (Kozhemyakina et al., 2016). Accordingly, the higher expression of Cacna1b in aggressive rats than in tame rats is probably associated with differences in anxiety-like behavior under novel conditions, as confirmed by the work of Kozhemyakina et al. (2016). In particular, in rats selected for increased aggressiveness, total motor activity for 5 min of the behavioral test is significantly higher; this parameter reflects the level of anxiety.

Our results somewhat contradict a study conducted on knockout mice, where it was shown that in the absence of calcium channel subunits, the aggressiveness of experimental animals is significantly higher (Kim C. et al., 2009). This discrepancy can be explained by the fact that the functioning of calcium channels is not directly related to aggressive reactions of the animal but rather is related to these reactions indirectly through a release of neurotransmitters, which, depending on the action of the neurotransmitter, determines the behavioral responses of the animal. For instance, serotonin, according to numerous studies, affects aggressiveness (Raleigh et al., 1991; Olivier, 2010), whereas the data on the correlation between serotonin levels and aggression (de Boer, Koolhaas, 2005) are contradictory. Achronic and sustained serotonin release is positively associated with both normal aggression (territorial conflicts or the establishment of a social hierarchy) (Raleigh et al., 1991; Audero et al., 2013) and with the pathological aggression characteristic of psychiatric patients (Zamponi, 2016). Thus, our study supplements the international research data on the relation between the expression of Cacna1b (encoding the calcium channel subunit) and aggressive behavior.

The expression of the Drd2 gene (dopamine D2 receptor) is associated with aggressive behavior, as uncovered in studies on rats (VanErp, Miczek, 2000) and on humans (Qadeer et al., 2017). Given that dopamine (an endogenous ligand [agonist] of D2 receptor), just as serotonin, is involved in the regulation of aggressive behavior, a change in Drd2 expression leads to various pathologies, for example, to increased aggressiveness (VanErp, Miczek, 2000; Miczek et al., 2002; Kim V. et al., 2015; Golden et al., 2019). At the same time, an aggressive interaction stimulates dopaminergic and serotonergic activities in the limbic regions of the brain (Summers, Winberg, 2006). In other words, hypothalamic-neuron activation, leading to the release of dopamine, may in turn promote the excitation of those hypothalamic neurons that control the attack (Yamaguchi, Lin, 2018). In relation to our study, these literature data indicate that the increased level of Drd2 expression in aggressive rats of both generations may actually be related to the phenotypic manifestation of aggressive reactions to humans.

The third differentially expressed gene in the rats selected for the reaction to humans, Egr1, encodes a transcription factor participating in the transcriptional activation of genes necessary for mitogenesis and cell differentiation. It is known that transcription factor Egr1 regulates the expression of genes that control synaptic plasticity and learning and memory processes; these functions make Egr1 an important object of research on the coherence of neural responses to various stimuli (Knapska, Kaczmarek, 2004). It has been reported that after exposure to stress, the expression of Egr1 in rats increases in neocortical regions, including the hypothalamus (Watanabe et al., 1994; Cullinan et al., 1995).

The higher expression of the Egr1 gene that we found in aggressive rats compared to tame rats can apparently be explained by the response to the stimulus (in the glove test, a human hand) that was employed for the artificial selection; in essence, this is a response to a stressor. Probably, in rats of the aggressive strain, the perception of the stimulus at the molecular level affects mechanisms of the genetic response to stress, in contrast to rats of the tame strain, which, as described above, react quite calmly not only to a human hand under the test conditions but also in general. Differential expression of Egr1 between the rats with genetically acquired aggressive or nonaggressive behavior toward humans is, in our opinion, an interesting result that can be applied to further research.

Gad2 is another gene for which we demonstrated differential expression between tame and aggressive rats of both generations. This gene encodes glutamate decarboxylase (GAD), which catalyzes the conversion of glutamate to GABA, a neurotransmitter that inhibits neuronal impulses. It is known that GABA controls aggressive behavior (Takahashi, Miczek, 2014; Hansen et al., 2018). Studies on mice have shown that aggressive animals have lower GABA levels due to decreased GAD activity in several regions of the brain (olfactory bulb, striatum, and amygdala) as compared to nonaggressive animals (Simler et al., 1982; Clement et al., 1987; Guillot, Chapouthier, 1998). On the other hand, these data were not confirmed in a study on Gad2 knockout mice, which have a reduced amount of GABA in the brain during postnatal development; however, such mutant males manifest reduced aggressiveness in the resident–intruder test (Stork et al., 2000). The effect of GABA depends on the area of the brain, the type of receptors, and the specific context of the situation causing the aggressive behavior (Takahashi, Miczek, 2014). In our work, the higher level of Gad2 expression in aggressive rats than in tame rats most likely corresponds to a situation when an increase in GABA synthesis in hypothalamic neurons causes an aggressive reaction of the animals in the “glove test,” which was employed for the artificial selection.

Furthermore, the factor analysis when the data on gene expression in the 88th and 90th generations were combined allows us to conclude that the following. Although the artificial selection was carried out by means of two vectors – (1) from the wild type to aggressive behavior and (2) from wild type to tame behavior – the expression of the 10 studied genes is associated with two factors: the difference between tame and aggressive rats (i. e., factor “domestication” because the selection for tame behavior is a model of domestication) and some general change that is the same for these two groups of animals (possibly the so-called laboratoryization effect, neutral drift, or something else). Meanwhile, the “domestication” factor is common between the rats of both generations but clearly distinguishes the animals by behavioral phenotype: tame or aggressive behavior (see Fig. 2). This result enables us to conclude that, indeed, the increased expression of genes Cacna1b, Drd2, Egr1, and Gad2 determines aggressive behavior in the selected rats, while the decreased expression corresponds to tameness.

Thus, genes Cacna1b, Drd2, Egr1, and Gad2, for which we showed interstrain differential expression in both generations (88th and 90th) of the rats selected for the reaction to humans, are promising for further studies on characteristics of domestication and aggressive behavior in animals. In our work, it was revealed that the manifestation of an aggressive and nonaggressive reaction to humans in rats of the 88th and 90th generations (of artificial selection for this trait) is controlled not by one but by several genes. Moreover, the protein products of these genes differ both in function and in the neurotransmitter systems in which they participate.

## Conclusion

Our expression analysis of 10 genes (by RT-PCR) in the hypothalamus of rats selected for a reaction to humans (tame and aggressive behavior) indicates that 4 genes are differentially expressed between tame and aggressive rats of both the 88th and 90th generation. Polygenic causes of the phenotypic manifestation of aggressive reactions were confirmed on model animals. Genes were identified that are most appealing for further research on the behavioral characteristics of rats selected for a response to humans.

## Conflict of interest

The authors declare no conflict of interest.
